# Implementation and clinical benefit of *DPYD* genotyping in a Danish cancer population

**DOI:** 10.1016/j.esmoop.2023.100782

**Published:** 2023-02-13

**Authors:** N.H. Paulsen, P. Pfeiffer, M. Ewertz, P.B.N. Fruekilde, S. Feddersen, H.S. Holm, T.K. Bergmann, C. Qvortrup, P. Damkier

**Affiliations:** 1 Department of Clinical Pharmacology, Odense University Hospital, Odense, Denmark; 2 Clinical Pharmacology, Pharmacy and Environmental Medicine, Department of Public Health, University of Southern Denmark, Odense, Denmark; 3 Department of Oncology, Odense University Hospital, Odense, Denmark; 4 Department of Clinical Research, University of Southern Denmark, Odense, Denmark; 5 Department of Clinical Biochemistry, Odense University Hospital, Odense, Denmark; 6 Department of Regional Health Research, University of Southern Denmark, Esbjerg, Denmark; 7 Department of Oncology, Rigshospitalet, University of Copenhagen, Copenhagen, Denmark

**Keywords:** DPD deficiency, *DPYD* genotype, DPD phenotype

## Abstract

**Background:**

In 2020, the European Medicines Agency recommended testing patients for dihydropyrimidine dehydrogenase (DPD) deficiency before systemic treatment with fluoropyrimidines (FP). DPD activity testing identifies patients at elevated risk of severe FP-related toxicity (FP-TOX). The two most used methods for DPD testing are *DPYD* genotyping and DPD phenotyping (plasma uracil concentration). The primary objective of this study was to compare the overall frequency of overall grade ≥3 FP-TOX before and after the implementation of *DPYD* genotyping.

**Patients and methods:**

Two hundred thirty Danish, primarily gastrointestinal cancer patients, were *DPYD*-genotyped before their first dose of FP, and blood was sampled for *post hoc* assessment of P-uracil. The initial dose was reduced for variant carriers. Grade ≥3 FP-TOX was registered after the first three treatment cycles of FP. The frequency of toxicity was compared to a historical cohort of 492 patients with *post hoc* determined *DPYD* genotype from a biobank.

**Results:**

The frequency of overall grade ≥3 FP-TOX was 27% in the *DPYD* genotype-guided group compared to 24% in the historical cohort. In *DPYD* variant carriers, *DPYD* genotyping reduced the frequency of FP-related hospitalization from 19% to 0%. In the control group, 4.8% of *DPYD* variant carriers died due to FP-TOX compared to 0% in the group receiving *DPYD* genotype-guided dosing of FP. In the intervention group, wild-type patients with uracil ≥16 ng/ml had a higher frequency of FP-TOX than wild-type patients with uracil <16 ng/ml (55% versus 28%).

**Conclusions:**

We found no population-level benefit of *DPYD* genotyping when comparing the risk of grade ≥3 FP-TOX before and after clinical implementation. We observed no deaths or FP-related hospitalizations in patients whose FP treatment was guided by a variant *DPYD* genotype. The use of DPD phenotyping may add valuable information in *DPYD* wild-type patients.

## Highlights

• No population-level benefit of *DPYD* genotyping was found.
• The use of DPD phenotyping may add valuable information in *DPYD* wild-type patients.
• No deaths or FP-related hospitalizations were seen in patients whose FP treatments were guided by a variant *DPYD* genotype.

## Introduction

Fluoropyrimidines (FP) are used to treat cancers of the gastrointestinal tract, head and neck, and breast. FP in Europe includes the marketed drugs 5-fluorouracil (5-FU) and its prodrugs capecitabine and tegafur. 5-FU is metabolized mainly by the rate-limiting dehydrogenase [dihydropyrimidine dehydrogenase (DPD)] enzyme.

Improving the safety of FP treatment using personalized medicine could impact millions of cancer patients treated worldwide for common cancers such as colorectal, upper gastrointestinal, pancreatic, and breast cancer. The most frequent uses of personalized medicine in oncology focus on improving the antitumor effect and overall survival of new targeted treatments. DPD testing has the potential to minimize the risk of toxicity associated with a widely used chemotherapeutic drug.

In 2020 the European Medicines Agency (EMA) recommended testing for DPD activity before treatment with FP to identify patients at increased risk of severe FP-related toxicity (FP-TOX).^1^ In a normal cancer population, the incidence of grade ≥3 [Common Terminology Criteria for Adverse Events (CTCAE)] FP-TOX is around 20%-30%.^2^^,^^3^ FP can, in rare cases, be lethal.^4^

The two most widely used methods to assess DPD activity are by genotyping the *DPYD* gene, which is the gene coding for the DPD enzyme, or measuring the endogenous metabolite uracil (DPD phenotype).

The *DPYD* genotyping test used in routine clinical practice typically examines four variants in the *DPYD* gene (*DPYD*∗2A, HapB3, D949V, *DPYD*∗13).^1^^,^^5^, ^6^, ^7^ The prevalence of these variants varies between 5% and 9% in a western European population but varies widely across the world.^8^

The plasma concentration of uracil is a surrogate marker for DPD activity because uracil is converted to dihydrouracil by the DPD enzyme.

Both testing strategies are used individually or combined in the routine treatment of cancer patients across Europe.^5^^,^^9^, ^10^, ^11^ The *DPYD* genotype and the DPD phenotype identify patients with partial or complete lack of DPD activity, as these patients are at elevated risk of developing FP-TOX if treated with full doses of FP. Currently, the most common strategy is to treat patients with partial DPD deficiency with a 50% reduced starting dose of FP and gradually increase the dose if there is no or only moderate toxicity. Administration of FP is not recommended if patients are compound heterozygous, but a phenotype test or genetic testing of the patients' parents may indicate whether the variants are located on the same allele, making them functionally as having partial DPD deficiency.^5^

Several studies have examined the clinical benefit of pretreatment DPD testing using *DPYD* genotyping and/or DPD phenotyping.^6^^,^^12^, ^13^, ^14^, ^15^, ^16^ These are very heterogeneous in design and do not provide a clear pattern as to the intervention’s best approach or clinical effect size. The most comprehensive study investigating *DPYD* genotyping was a prospective analysis from the Netherlands, including 1103 patients.^6^ All patients received pretreatment *DPYD*-guided FP dosing, and the frequency of FP-TOX was compared to a historical cohort. The study showed a clear reduction of FP-TOX in *DPYD* variant carriers who received pretreatment FP dose reductions.

The primary objective of the study was to compare the frequency of overall grade ≥3 FP-TOX before and after clinical implementation of pretreatment *DPYD* genotyping for the four most common variants. Secondary endpoints included the frequency of FP-related hospitalizations, FP-related death, and discontinuation of FP due to toxicity.

## Methods

### Study design

This study was a prospective single-center analysis of cancer patients with a historic group as controls (ClinicalTrials.gov identifier: NCT05266300). All patients were treated at the Department of Oncology, Odense University Hospital (OUH), Denmark.

The intervention group received pretreatment *DPYD* genotyping before FP treatment, whereas the control group was treated before *DPYD* genotype implementation. The *DPYD* genotype in the control group was analyzed later using biobank material. Patients in the intervention group had blood samples collected in a biobank for later uracil measurements.

Patients in the intervention group were enrolled from 1 September 2020, i.e. shortly after the *DPYD* genotype was implemented as a standard of care on 1 July 2020, to 31 December 2021. The study was planned and approved before the recommendation from EMA in the spring of 2020.^1^

Inclusion criteria were patients planned for their first systemic treatment with 5-FU, capecitabine, or tegafur, regardless of the tumor type.

Patients not included in the study’s prospective part (intervention group) were still genotyped as it was standard at the department at the time and received doses according to the *DPYD* variants. All patients in the intervention group were *DPYD*-genotyped before receiving their first dose of FP. Patients were categorized as *DPYD* wild type if none of the tested *DPYD* variants (*DPYD*∗2A, HapB3, D949V, *DPYD*∗13) were identified.

### Sample-size

Based on reported frequencies of grade 3 or higher toxicity among untested patients and variant carriers, and assuming a cumulative minor allele frequency of 7%, we estimated that 1000 patients in the intervention population and 2000 in the control group would allow for the detection of a 25% reduction in overall grade ≥3 FP-TOX in the entire population with a power of 80% and a significance level of 5%.

### Treatment algorithm

The dose reduction algorithm used included a 50% dose reduction of the planned FP dose in patients who were heterozygous for one of the tested variants.^17^ A careful and graduate dose escalation (most often to 75% and further to 100%) was recommended in patients tolerating the 50% dose without moderate or severe toxicity. FP treatment was not recommended in compound heterozygous (carriers of two or more different *DPYD* variants) or homozygous patients for the tested variants.

### Intervention group (DPYD guided)

Participation in the study included blood samples for measuring the uracil concentration and permission for the researchers to collect health data from their electronic health records and registries.

The study was approved by the local ethics committee and followed the Declaration of Helsinki. Written and informed consent was collected from all participants. The study protocol was approved by The Regional Committees on Health Research Ethics for Southern Denmark (H-B-2008-037).

### Control group

The control group was treated with their first dose of FP between 1 June 2017 and 30 June 2020 at the Department of Clinical Oncology at OUH and where biobank material was available for *DPYD* genotype analysis from the Danish CancerBiobank.^18^ Permission to analyze the samples collected from the Danish CancerBiobank and to collect data from electronic health records without written consent from the participants was granted by The Regional Committees on Health Research Ethics for Southern Denmark (H-B-2008-037).

### DPYD genotyping and DPD phenotyping (plasma uracil measurements)

*DPYD* genotyping was done using the LAMP Human DPD deficiency KIT (LaCAR MDx Technologies, Liège, Belgium) and real-time PCR. Liquid chromatography-tandem mass spectrometry was used in the analysis of the plasma uracil levels. These methods have been described earlier.^7^ For more details, see Supplementary Appendix S1, available at https://doi.org/10.1016/j.esmoop.2023.100782. Samples for the determination of uracil concentration were analyzed *post hoc*.

### Collection of clinical data

All data collection was done after patients in both groups had been included and completed up to four treatment cycles of FP. Data were collected from different sources, merged using the patient’s social security number, and stored on a secure server for analysis.

#### Chemotherapy

Data describing the dose, date, and specific chemotherapy used were collected from the hospital pharmacy at OUH.

#### Blood samples

Data from all clinical blood samples analyzed doing treatment in the Region of Southern Denmark were collected and stored. Data were also used to screen if patients suffered hematological toxicity, such as thrombocytopenia and neutropenia.

#### Comorbidity

Information regarding the patients’ diseases other than cancer was collected from the patients’ health register.

#### Clinical data

Data regarding patients’ performance status (World Health Organization), tumor type, FP-related admissions, FP-associated toxicity, toxicity type, discontinuation of FP drugs due to FP toxicity, reasons for dose-adjusting FP, treatment intensity, and treatment goal were collected from patients’ electronic health records.

### Outcomes

Toxicity outcomes were collected and registered after the first three cycles of FP treatment if available. We chose only to include the first three cycles as severe FP-TOX will likely be detected during the first treatment cycles.

### Toxicity scoring

The primary outcome of this study was the incidence of FP-associated grade ≥3 toxicity rated according to CTCAE v5.0.^19^ Secondary outcomes were the incidence of FP-related hospitalization, FP-related death, and discontinuation of FP due to toxicity.

All toxicity scoring was done retrospectively using data from chemotherapy dosing, blood samples, and the patient’s electronic health records. Eighty percent of patients received combination chemotherapy, and only toxicity events evaluated as being induced by FP were scored and registered. A toxicity event in patients receiving combination therapy was evaluated as being FP induced if the adverse events were probably related to FP according to the most common adverse events known for the specific drugs used.

Adverse events were merged into the following categories: gastrointestinal, hematological, cardiac, palmar plantar erythrodysesthesia (PPE), and others.

A physician (NHP) scored all patients for FP-TOX under supervision from a senior consultant in clinical oncology (PP).

### Statistics

Data are described using percentage distributions, means, and standard deviations or medians and ranges as appropriate. The relative risk (RR) reported is defined as the ratio of the FP-TOX risk in patients carrying *DPYD* variants or uracil ≥16 ng/ml compared to wild type or normal uracil. Data handling, production of plots, and statistical analysis were carried out using Stata/SE version 17 and R version 4.1.1.

## Results

In total, 239 patients were enrolled in the intervention group between 1 September 2020 and 31 December 2021. Seven patients never received treatment with FP and were therefore excluded. Two patients had previously received FP treatment and were excluded (Supplementary Figure S1, available at https://doi.org/10.1016/j.esmoop.2023.100782). No homozygous patients for any *DPYD* variants were found in the intervention group.

Two patients had very high uracil levels (251 and 267 ng/ml), and levels of 5-FU were found in the samples. These samples were excluded because patients were in active treatment at the sampling time.

Of the 230 patients who gave consent and were treated with FP, 202 had blood samples collected for uracil analysis. Two hundred of the samples were included in the analysis.

In the control group, 498 patients were treated with FP in the inclusion period (1 June 2017 to 30 June 2020) and had blood samples available in the Danish CancerBiobank.

The baseline characteristics of patients in the intervention and control groups are shown in Table 1. The two groups of patients were comparable with respect to age, sex, cancer diagnosis, performance status, and anticancer drugs. The distribution of *DPYD* variants was similar in the two groups. A larger part of patients in the control group were treated in a curative setting (70%) compared to the intervention group (53%).

**Table 1:** Baseline characteristics of 722 cancer patients treated at Odense University Hospital

	Overall *N* = 722^a^	Intervention (*DPYD* guided) *n* = 230^a^	Control *n* = 492^a^
Sex			
Male	456 (63)	137 (60)	319 (65)
Female	266 (37)	93 (40)	173 (35)
Age at inclusion, years	66.7 (9.4)	66.8 (10.0)	66.7 (9.1)
Tumor type			
Upper gastrointestial (adenocarcinoma)	221 (31)	54 (23)	167 (34)
Colorectal	195 (27)	66 (29)	129 (26)
Pancreas	138 (19)	44 (19)	94 (19)
Bile duct	61 (8.4)	23 (10)	38 (7.7)
Breast	43 (6.0)	27 (12)	16 (3.3)
Esophageal (squamous cell carcinoma)	26 (3.6)	7 (3.0)	19 (3.9)
Head and neck	16 (2.2)	0 (0)	16 (3.3)
Duodenal cancer	13 (1.8)	5 (2.2)	8 (1.6)
Neuroendocrine carcinoma	7 (1.0)	3 (1.3)	4 (0.8)
Carcinoma of unknown primary	2 (0.3)	1 (0.4)	1 (0.2)
First treatment with fluoropyrimidine			
S-1 [teysuno, (tegafur)]	321 (44)	92 (40)	229 (47)
Capecitabine	271 (38)	97 (42)	174 (35)
5-FU	130 (18)	41 (18)	89 (18)
Number of treatment cycles with FP			
4	575 (80)	175 (76)	400 (81)
3	66 (9.1)	22 (9.6)	44 (8.9)
2	38 (5.3)	14 (6.1)	24 (4.9)
1	43 (6.0)	19 (8.3)	24 (4.9)
First FP dose intensity, %			
100	607 (84)	167 (73)	440 (89)
51-85	92 (13)	41 (18)	51 (10)
50	22 (3.1)	21 (9.2)	1 (0.2)
<50	1 (0.1)	1 (0.4)	0 (0)
Treatment target			
Curative	469 (65)	123 (53)	346 (70)
Palliative	253 (35)	107 (47)	146 (30)
Performance status (WHO)			
0	347 (48)	110 (48)	237 (48)
1	305 (42)	94 (41)	211 (43)
2	69 (9.6)	26 (11)	43 (8.7)
3	1 (0.1)	0 (0)	1 (0.2)
*DPYD* genotype			
Wild type	658 (91)	207 (90)	450 (91)
*DPYD* variants			
Heterozygous (HapB3)	39 (5.4)	12 (5.2)	27 (5.5)
Heterozygous (D949V)	9 (1.1)	5 (2.2)	4 (0.8)
Heterozygous (*DPYD*∗2A)	8 (1.1)	1 (0.4)	7 (1.4)
Heterozygous (*DPYD∗*13)	4 (0.6)	3 (1.3)	1 (0.2)
Homozygous			
HapB3/HapB3	2 (0.3%)	0 (0%)	2 (0.4%)
Compound heterozygous			
D949V and HapB3	1 (0.1%)	1 (0.4%)	0 (0%)
*DPYD*2∗Aand HapB3	1 (0.1%)	0 (0%)	1 (0.2%)
Comorbidity			
Chronic obstructive pulmonary disease	24 (3.3%)	6 (2.6%)	18 (3.7%)
Type 1 diabetes	14 (1.9%)	6 (2.6%)	8 (1.6%)
Type 2 diabetes	84 (12%)	25 (11%)	59 (12%)
eGFR interval (ml/min/1.73 m^2^)			
>90	291 (42)	98 (44)	193 (41)
60-89	338 (49)	105 (47)	233 (0)
30-59	63 (9.1)	21 (9.4)	42 (9.0)
<30	0 (0)	0 (0)	0 (0)
Unknown	30	6	24
Radiotherapy during FP treatment	49 (6.8)	12 (5.2)	37 (7.5)
Combination chemotherapy	577 (80)	179 (78)	398 (81)
Other drugs during first treatment cycle			
Oxaliplatin	397 (55)	122 (53)	275 (56)
Docetaxel	137 (19)	25 (11)	112 (23)
Gemcitabine	144 (20)	54 (23)	90 (18)
Calcium folinate(folinic acid)	105 (15)	35 (15)	70 (14)
Bevacizumab	9 (1.2)	4 (1.7)	5 (1.0)
Cisplatin	11 (1.5)	1 (0.4)	10 (2.0)
Cyclophosphamide	3 (0.4)	1 (0.4)	2 (0.4)
Irinotecan	64 (8.9)	23 (10)	41 (8.3)
Methotrexate	3 (0.4)	1 (0.4)	2 (0.4)
Cetuximab	15 (2.1)	5 (2.2)	10 (2.0)
Paclitaxel	18 (2.5)	0 (0)	18 (3.7)
Pembrolizumab	1 (0.1)	1 (0.4)	0 (0)
Temozolomide	3 (0.4)	2 (0.9)	1 (0.2)
Uracil concentration (ng/ml)		*n* = 200	
Mean uracil concentration (SD)		8.9 (4.1)	
<16		187 (94)	
≥16 <150		12 (6.0)	

FP, (5-fluorouracil, capecitabine, tegafur(S-1));

eGFR, estimated glomerular filtration rate; 5-FU, 5-fluoruracil; SD, standard deviation; WHO, World Health Organization.

a *n* (%).

All 22 patients carrying *DPYD* variants in the intervention group received reduced doses of FP according to the treatment guideline. A dose escalation was carried out in 13 of the 22 patients (59%). Escalations were attempted and abandoned in two additional patients due to toxicity.

### Comparison of FP-TOX between the intervention group and control group

No difference in overall grade ≥3 FP-TOX between the two groups was found, with 24% in the control group and 27% in the intervention group, respectively (Table 2).

**Table 2:** Frequency of fluoropyrimidine-related toxicity in the two groups split into wild type and all *DPYD* variant carriers

	Intervention group (*DPYD* guided)^a^			Control group^a^		
	Overall, *N* = 230	*DPYD* variant, *n* = 22	Wild type, *n* = 208	Overall, *N* = 492	*DPYD* variant, *n* = 42	Wild type, *n* = 450
Relative dose intensity first cycle^b^	91 (38-100)	49 (38-50)	96 (75-100)	98 (50-100)	97 (75-100)	98 (50-100)
Overall grade ≥3 toxicity	63 (27)	5 (23)	58 (28)	112 (23)	12 (29)	100 (22)
Stop of FP treatment due to toxicity	14 (6.1)	0	14 (6.7)	30 (6.1)	3 (7.1)	27 (6.0)
FP-related hospitalization	23 (10)	0	23 (11)	40 (8.1)	8 (19)	32 (7.1)
Worst FP grade registered						
Grade 3 toxicity	56 (24)	5 (23)	51 (25)	94 (19)	7 (17)	87 (19)
Grade 4 toxicity	6 (2.6)	0	6 (2.9)	12 (2.4)	3 (7.1)	9 (2.0)
Grade 5 toxicity (death)	1 (0.4)	0	1 (0.5)	6 (1.2)	2 (4.8)	4 (0.9)
Grade ≥3 gastrointestinal toxicity	34 (15)	3 (14)	31 (15)	60 (12)	9 (21)	51 (11)
Grade ≥3 haematological toxicity	11 (4.8)	0	11 (5.3)	26 (5.3)	4 (9.5)	22 (4.9)
Grade ≥3 cardiac toxicity	8 (3.5)	1 (4.5)	7 (3.4)	10 (2.0)	0	10 (2.2)
Grade 3 PPE	9 (3.9)	1 (4.5)	8 (3.8)	14 (2.8)	1 (2.4)	13 (2.9)
Grade ≥3 Other toxicity	2 (0.9)	0	2 (1.0)	2 (0.4)	0	2 (0.4)

Heterozygous, compound heterozygous, and homozygous are pooled together. FP, (5-fluorouracil, capecitabine, tegafur(S-1));

PPE, palmar plantar erythrodysesthesia.

a *n* (%).

b Mean in % of normal dose of FP drug relative to regimen (min-max), in % (min-max); all toxicity reported in this table is related to FP.

### *Toxicity in patients carrying* DPYD *variants*

Reduction in FP-related hospitalizations was seen when comparing patients carrying *DPYD* variant in the intervention group and the control group; 0/22 (0%) versus 8/42 (19%) [RR 0.11, 95% confidence interval (CI) 0.01-1.82] (Tables 2 and 5).

No patients receiving pretreatment *DPYD*-guided dosing suffered from FP-related death or were hospitalized because of FP-TOX. In the control group, two patients (4.8%) died due to FP-TOX, and eight (19%) were admitted to the hospital due to FP-TOX.

### *Specific* DPYD *variants*

Of the seven patients with the *DPYD*∗2A variant in the control group, two patients (29%) died due to FP-TOX, and 71% (5/7) suffered grade ≥3 FP-TOX. One patient in the control group was compound heterozygous with the *DPYD*∗2A and HapB3 variants, suffered from grade 4 FP-TOX, and was hospitalized (Table 3).

**Table 3:** Frequency of fluoropyrimidine-related toxicity in the two groups split into wild type and specific *DPYD* variants

	Intervention group (*DPYD* guided)^a^						Control group^a^						
	CH (D949V, HapB3), *n* = 1	HT (D949V), *n* = 5	HT (*DPYD*13), *n* = 3	HT (*DPYD*2A), *n* = 1	HT (HapB3), *n* = 12	Wild type, *n* = 208	CH (DPYD2A-HapB3), *n* = 1	HT (D949V), *n* = 4	HT (*DPYD*13), *n* = 1	HT (*DPYD*2A), *n* = 7	HT (HapB3), *n* = 27	HO (HapB3), *n* = 2	Wild type, *n* = 450
Relative dose intensity first cycle^b^	50 (50-50)	50 (50-50)	50 (50-50)	50 (50-50)	49 (38-50)	96 (75-100)	100 (100-100)	94 (75-100)	80 (80-80)	100 (100-100)	98 (75-100)	100 (100-100)	98 (50-100)
Overall grade ≥3 toxicity	0	1 (20)	1 (33)	1 (100)	2 (17)	58 (28)	1 (100)	0	0	5 (71)	6 (22)	0	100 (22)
Stop of FP treatment due to toxicity	0	0	0	0	0	14 (6.7)	1 (100)	0	0	1 (14)	1 (3.7)	0	27 (6.0)
FP-related hospitalization	0	0	0	0	0	23 (11)	1 (100)	0	0	5 (71)	2 (7.4)	0	32 (7.1)
Worst FP grade registered.													
Grade 3 toxicity	0	1 (20)	1 (33)	1 (100)	2 (17)	51 (25)	0	0	0	2 (29)	5 (19)	0	87 (19)
Grade 4 toxicity	0	0	0	0	0	6 (2.9)	1 (100)	0	0	1 (14)	1 (3.7)	0	9 (2.0)
Grade 5 toxicity (death)	0	0	0	0	0	1 (0.5)	0	0	0	2 (29)	0	0	4 (0.9)
Grade ≥3 gastrointestinal toxicity	0	0	0	1 (100)	2 (17)	31 (15)	0	0	0	4 (57)	5 (19)	0	51 (11)
Grade ≥3 hematological toxicity	0	0	0	0	0	11 (5.3)	1 (100)	0	0	2 (29)	1 (3.7)	0	22 (4.9)
Grade ≥3 cardiac toxicity	0	1 (20)	0	0	0	7 (3.4)	0	0	0	0	0	0	10 (2.2)
Grade 3 PPE	0	0	1 (33)	0	0	8 (3.8)	0	0	0	0	1 (3.7)	0	13 (2.9)
Grade ≥3 other toxicity	0	0	0	0	0	2 (1.0)	0	0	0	0	0	0	2 (0.4)

FP, (5-fluorouracil, capecitabine, tegafur(S-1));

PPE, palmar plantar erythrodysesthesia; CH, compound heterozygous; HT, heterozygous; HO, homozygous.

a *n* (%).

b Mean in % of normal dose of FP drug relative to regimen. (min-max); all toxicity reported in this table is related to FP.

Patients carrying the most common *DPYD* variant HapB3 in the control group had similar frequencies of FP-TOX compared to wild-type patients across both groups.

Two patients were identified as homozygous for the HapB3 variant in the control group. Both patients were treated with a 100% starting dose, but none experienced grade ≥3 FP-TOX.

One patient was compound heterozygous in each group. The patient in the intervention group carried the variants HapB3 and *DPYD*∗2A. The responsible senior physician requested a uracil measurement (9.5 ng/ml), and the patient subsequently received 50% S-1 + oxaliplatin (SOX). The patient tolerated the treatment well and was escalated to 75% during the second treatment cycle and to 100% during the third cycle, respectively.

The heterozygous patient in the control group carried the *DPYD*∗2 and HapB3 variants. This patient was treated with a 100% starting dose of the CAPOX (capecitabine + oxaliplatin) regimen and experienced neutropenic fever leading to hospitalization.

### DPD phenotype (plasma uracil concentration)

Thirteen (6.5%) of the 200 available patient samples had uracil concentration values of ≥16 ng/ml and <150 ng/ml, categorizing them as having partial DPD deficiency.^1^ No values above 150 ng/ml indicative of complete DPD deficiency were identified. Eleven patients with elevated uracil concentration (≥16 ng/ml, <150 ng/ml) were classified as *DPYD* wild type and were thus treated with standard doses of FP (Supplementary Table S1, available at https://doi.org/10.1016/j.esmoop.2023.100782). These 11 patients experienced a higher frequency of FP-TOX compared to *DPYD* wild-type patients with normal levels of uracil (5.8% versus 55%).

**Figure 1:**
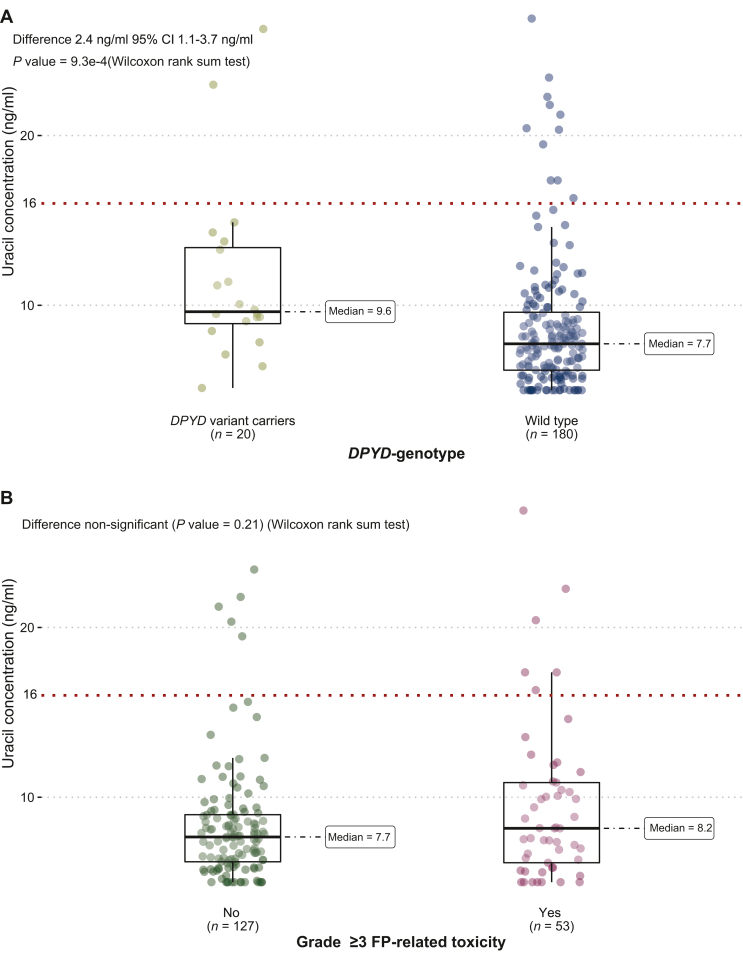
**Uracil concentration distribution**. (A) Uracil concentration distribution compared between wild type and *DPYD* variant carriers (heterozygous, compound heterozygous, and homozygous are pooled together). (B) Uracil concentration of wild-type patients with grade ≥3 FP-TOX compared to wild-type patients without toxicity. FP, (5-fluorouracil, capecitabine, tegafur(S-1));

Two patients in the *DPYD* variant group had uracil values above 16 ng/ml (Figure 1A). One patient carried the variant *DPYD*∗13A with P-uracil at 26.3 ng/ml and experienced grade 3 FP-TOX (PPE) when the dose of capecitabine was escalated to 100% during the third cycle.

One patient in the intervention group died due to FP-TOX. This patient had normal uracil concentrations (5.6 ng/ml) and was *DPYD* wild type. The patient died after the first treatment cycle with SOX in 100% dosing (grade 5 hematological toxicity).

The strength of the correlation between uracil concentration in the *DPYD* variant compared to wild type was poor. In Figure 1A, the distribution of uracil is plotted in patients being wild type and patients carrying *DPYD* variants. In Figure 1B, the uracil concentration of wild-type patients suffering from FP-TOX is compared to wild-type patients without overall grade ≥3 FP-TOX.

### Risk of toxicity

Table 4 illustrates the RR of grade ≥3 FP-TOX in patients carrying *DPYD* variants compared with wild-type patients. Genotype-guided dosing suggested a potential reduction in the RR of grade ≥3 FP-TOX in *DPYD* variant carriers from 1.29 (95% CI 0.77-2.2) in the control group to 0.82 (95% CI 0.37-1.8) in patients who received a 50% starting dose.

**Table 4:** Relative risk of grade ≥3 FP-related toxicity in *DPYD* variant carriers compared with wild type and in patients with elevated uracil concentrations (≥16 ng/ml) compared with normal values

*DPYD* genotype		
Relative risk versus wild type in each group	Intervention (*DPYD*-guided doses)	Control group (normal dose)
*DPYD*∗2A^a^	1 patient (grade 3 tox)	3.42 (2.2-5.29) (*n* = 8)
*DPYD*∗13	0.55 (0.15-2.00) (*n* = 3)	1 patient (no tox)
D949V	0.60 (0.10-3.65) (*n* = 6)	0.44 (0.03-6.11) (*n* = 3)
HapB3^b^	0.55 (0.15-1.99) (*n* = 13)	1.02 (0.53-2.01) (*n* = 30)
All *DPYD* variants	0.82 (0.37-1.82) (*n* = 22)	1.29 (0.77-2.18) (*n* = 42)

Uracil intervention group			
**Patients with ≥16 ng/ml versus normal uracil**	**All patients, *n* = 200**	**Wild-type patients (normal dose), *n* = 180**	***DPYD* variants (reduced doses) FP, *n* = 20**
≥16 ng/ml	1.97 (1.13-3.44) (*n* = 13)	1.96 (1.09-3.54) (*n* = 11)	2.25 (0.44-11.52) (*n* = 2)
>150 ng/ml	No patients found		

All data reported with 95% confidence intervals.

FP, (5-fluorouracil, capecitabine, tegafur(S-1)); Tox, toxicity.

a Includes one patient compound heterozygous for *DPYD*∗2A and HapB3.

b Includes two patients homozygous for HapB3 and one patient compound heterozygous for *DPYD∗*2A and HapB3.

Only one of the tested *DPYD* variants showed a significant increase in the RR of toxicity in the control group despite being treated with normal doses. The variant *DPYD*∗2A carriers in the control group had a statistically significantly increased risk of overall grade ≥3 FP-TOX compared to wild-type patients [RR 3.42 (95% CI 2.2-5.3)].

Table 5 illustrates FP-TOX risk between the intervention and the control group. For *DPYD* variant carriers, the risk of FP-related hospitalization was 0.11 (95% CI 0.01-1.8) in the intervention group compared to the control group.

**Table 5:** Relative risk of FP-related toxicity in the intervention group compared to control group

	All patients	*DPYD* variant carriers	Wild type
Overall grade ≥3 toxicity	1.20 (0.92-1.57)	0.80 (0.32-1.97)	1.25 (0.95-1.66)
FP-related hospitalization	1.23 (0.75-2.00)	0.11 (0.01-1.82)	1.56 (0.93-2.59)
Grade 5 toxicity (death)	0.36 (0.04-2.94)	0.37 (0.02-7.46)	0.54 (0.06-4.81)

All data reported with 95% confidence intervals.

FP, (5-fluorouracil, capecitabine, tegafur(S-1))

Heterozygous, compound heterozygous, and homozygous are pooled together.

Patients with uracil contractions ≥16 ng/ml had significantly increased RR of overall grade ≥3 FP-TOX of 2.0 (95% CI 1.1-3.4) compared to patients with normal levels of uracil. When only wild-type patients were treated with normal doses of FP, the RR was 2.0 (95% CI 1.1-3.5) (Table 4).

### Specific FP drugs

Overall, no apparent difference in the incidence of overall FP-TOX was seen between the three different FP drugs (Supplementary Tables S2 and S3, available at https://doi.org/10.1016/j.esmoop.2023.100782).

## Discussion

In this study, we found no population-level effect on the incidence of FP-related severe toxicity when comparing preemptive *DPYD* genotyping against standard treatment in a historical control cohort.

We observed no deaths or FP-related hospitalizations in patients whose FP treatment was guided by a variant *DPYD* genotype. On the contrary, in the control group, where *DPYD* variant carriers received standard doses of FP, 2 of 42 patients (4.8%) died due to FP-TOX, and 8 (19%) were hospitalized due to FP-TOX.

Of the *DPYD* variants examined, only one (*DPYD*∗2A) was associated with a statistically significantly more than threefold increased risk of grade ≥3 FP-TOX compared to wild-type patients (RR 3.4, 95% CI 2.2-5.3). Finally, patients in the intervention group with uracil concentrations ≥ 16 ng/ml had a statistically significantly increased risk of grade ≥3 FP-TOX (RR 2.0, 95% CI 1.1-3.4) compared to patients with normal levels of uracil.

This clinical study was planned and designed before the EMA^1^ recommended DPD enzyme activity testing to all patients before treatment with FP. The recommendation did not alter the original planned design, as we found that a randomized setup would be unethical, given the evidence available at the time. Given the totality of the evidence at the time, there was not a reasonable degree of equipoise between intervention and treatment as usual.^6^^,^^13^

The prevalence of clinically relevant *DPYD* variants in the study population was 8.7%, which is in line with other European studies^6^^,^^20^ and Denmark.^7^ This highlights that these variants are relevant in the Danish population but may not be relevant to other populations.^8^

The correlation between *DPYD* variant carriers and uracil concentration was unconvincing, and the population difference in the median uracil concentration between wild type and *DPYD* variant carriers was small and unlikely to be of clinical difference (9.6 ng/ml versus 7.7 ng/ml). These findings compare well to other European^20^^,^^21^ and Danish studies.^7^

### Toxicity

The study failed to show an overall benefit of *DPYD* genotyping in the intervention group. The overall incidence of overall grade ≥3 FP-TOX was 27% in the intervention group compared to 23% in the control group. FP-related death was 0.4% in the intervention group compared to 1.2% in the control group.

When focusing only on patients carrying the tested *DPYD* variants in the two groups, the use of DPD testing reduced the risk of grade 4 FP-TOX, FP-related hospitalization, and FP-related death to 0% (*n* = 22). The frequency of overall grade ≥3 FP-TOX was similar in the two groups, with 23% in the intervention group compared to 29% in the control group.

When comparing the risk of FP-TOX in the specific *DPYD* variant carriers in the control group, patients carrying the *DPYD*∗2A had the highest risk of death and severe toxicity (Table 3).

No FP-TOX was seen for patients heterozygous for the *DPYD*∗13 variant (*n* = 1) and D949V (*n* = 4) variant in the control group, even though they received standard doses of FP. Given the small sample size, no clinical inferences can be made from these observations.^6^^,^^13^ As the phenotype–genotype correlation is poor, e.g. (heterozygote) variant genotype carriers often demonstrate normal phenotype as assessed by P-uracil, this reduces the ability to predict toxicity.^8^ From Figure 1B—only wild-type patients—and Supplementary Figures S2-S4 and , available at https://doi.org/10.1016/j.esmoop.2023.100782 (all genotyped patients and only variant carrying patients), it may be hypothesized that phenotyping is a better predictor of toxicity as 6/53 (7/58 among all genotyped) with toxicity had a uracil value above 16 ng/ml compared to 5/127 (6/142 among all genotyped) who did not have toxicity. Contrarily, however, 5/6 patients with increased P-uracil did not have toxicity while 47/53 (51/58) patients with normal P-uracil did have toxicity. The latter observations complicate the interpretation of the genotype–phenotype–toxicity associations. Overall, our findings do not allow to prioritize between the two approaches.

### HapB3 variant

The HapB3 (*n* = 27) variant was the most dominant variant (5.5%), and the frequency of FP-TOX was very similar to wild-type patients in the control group (Table 3).

Patients heterozygous for the HapB3 variant were earlier recommended treatment with a 25% dose reduction^22^ which was later changed to 50% after a prospective clinical study from the Netherlands.^6^ Studies have shown that DPD enzyme activity determined in peripheral blood mononuclear cells was very similar in HapB3 carriers and wild-type patients.^21^^,^^23^ The same trend is seen when comparing the uracil concentration in HapB3 patients versus wild type.^7^^,^^21^ A study from 2021 also found that HapB3 carriers (*n* = 41) treated without dose reductions had similar toxicity compared to wild-type patients (grade ≥3 FP-TOX 34% in HapB3 carriers versus 31% in wild-type patients).^16^ Another study did not show elevated toxicity risk of HapB3 carriers treated with normal doses of FP with a reported adjusted odds ratio of 1 (0.55-1.9).^15^ In a meta-analysis, the HapB3 variant was the only variant that was not associated with an increased risk of FP-related death compared to wild-type patients [risk of death 0.4 (0.1-2.9)]. One patient of 241 HapB3 carriers died due to FP-related toxicity.^4^

These results align with the similar incidence of FP-TOX in HapB3 carriers compared to the wild type found in the control group of this study. Because the HapB3 variant is the most common variant of the tested, it will significantly lower the clinical impact of *DPYD* genotyping if patients with HapB3 are not DPD deficient. If patients with the HapB3 variant do not have a clinically relevant decrease in their DPD enzyme activity, this may lead to underdosing and poor treatment outcomes.

### Uracil concentration

Our results indicate that DPD phenotyping may include additional information on identifying patients with partial DPD deficiency. Wild-type patients with high uracil had a higher risk of grade ≥3 FP-TOX [RR 2.0 (1.1-3.5)].

The correlation between P-uracil, *DPYD* variant carriers, and wild-type patients was poor (Figure 1A). This level of correlation is in line with other studies in European populations.^7^^,^^20^^,^^21^ The poor correlation between the two tests does not, by default, mean that the uracil measurement is better than *DPYD* genotyping. The current evidence supporting the phenotype and the current cut-off values is insufficient; so concluding that phenotyping is superior to genotyping is premature.^12^^,^^21^ The measurement of plasma uracil has some drawbacks compared to *DPYD* genotyping. The plasma concentration is affected by food intake, circadian rhythm, and severe renal impairment^24^, ^25^, ^26^ and is sensitive to preanalytical variation if the samples are not handled correctly.^27^

### S-1

This study included 320 patients treated with the DPD enzyme inhibitor (gimeracil) as part of S-1, which may reduce the impact of patients’ DPD enzyme activity.^28^ Our results for S-1 show that the frequency of overall FP-TOX was similar across the three different FP drugs.

### Strengths

As part of the clinical study, we implemented the *DPYD* genotype as a new standard of care. The clinical implementation was successful, and all patients with *DPYD* variants were treated with the correct reduced dose of FP. Dose escalation was attempted in 59% of the variant carriers. This study was carried out in a routine clinical setting, reflecting the real-life benefit of DPD testing in an unselected group of cancer patients. The characteristics of patients in the control group used were similar to those in the intervention group. The control group included patients treated in the same department from the 3 years preceding implementation mitigating possible changes to clinical practice.

### Limitations

Due to COVID-19-related issues, we could only recruit 230 patients in the intervention group and we only included dose intervention in 22 *DPYD* variant patients. This is substantially below our prespecified sample size and, consequently, severely reduces our ability to detect clinically relevant differences.

The study only examined the four most common *DPYD* variants causing DPD deficiency. Whole *DPYD* gene sequencing may have revealed more patients carrying clinically relevant *DPYD* mutations. No pharmacokinetic information was collected to examine *DPYD* variant carriers’ exposure to FP when treated with reduced doses of FP.

The study did not examine patients’ survival or response rate and cannot answer if patients treated with reduced doses were insufficiently treated with FP drugs.

### Conclusion

The study could not demonstrate a population-level reduction in the frequency of overall grade ≥3 FP-TOX after *DPYD* genotype implementation.

Subgroup analysis suggests that focused assessment of *DPYD* variant carriers potentially reduces hospitalizations and death among cancer patients needing FP treatment. We observed no deaths or FP-related hospitalizations in patients whose FP treatment was guided by variant *DPYD* genotype.

*DPYD* variant carriers treated with full doses of S-1 had similar FP-TOX rates compared to capecitabine and 5-FU.

Further studies should examine other methods for identifying DPD enzyme deficiency, including DPD phenotyping, which may be superior to genotyping.
