# Supplementary figures and images for: Implementation and clinical benefit of DPYD genotyping in a Danish cancer population
Source: ESMO Open. 2023 Feb 13;8(1):100782. doi: 10.1016/j.esmoop.2023.100782 (PMC10024141; doi:10.1016/j.esmoop.2023.100782)

**Figure S1.** Overview of included patients in the study. All patients was treated in Odense, Denmark

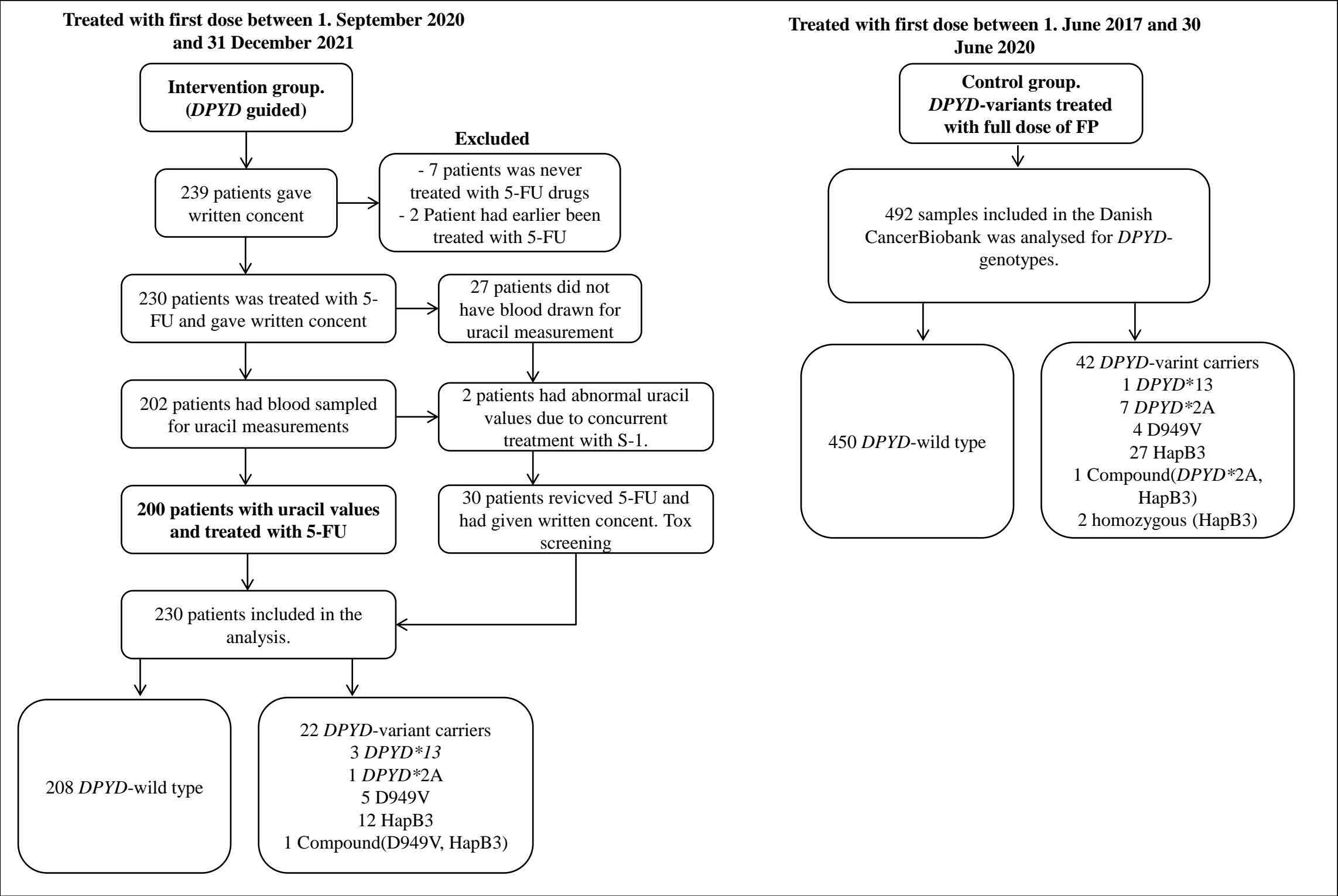

Supplement: Supplementary Figure S1 [file mmc1.pdf]

**Figure S4.** Uracil distribution across specific *DPYD*-variants carriers and wild type patients

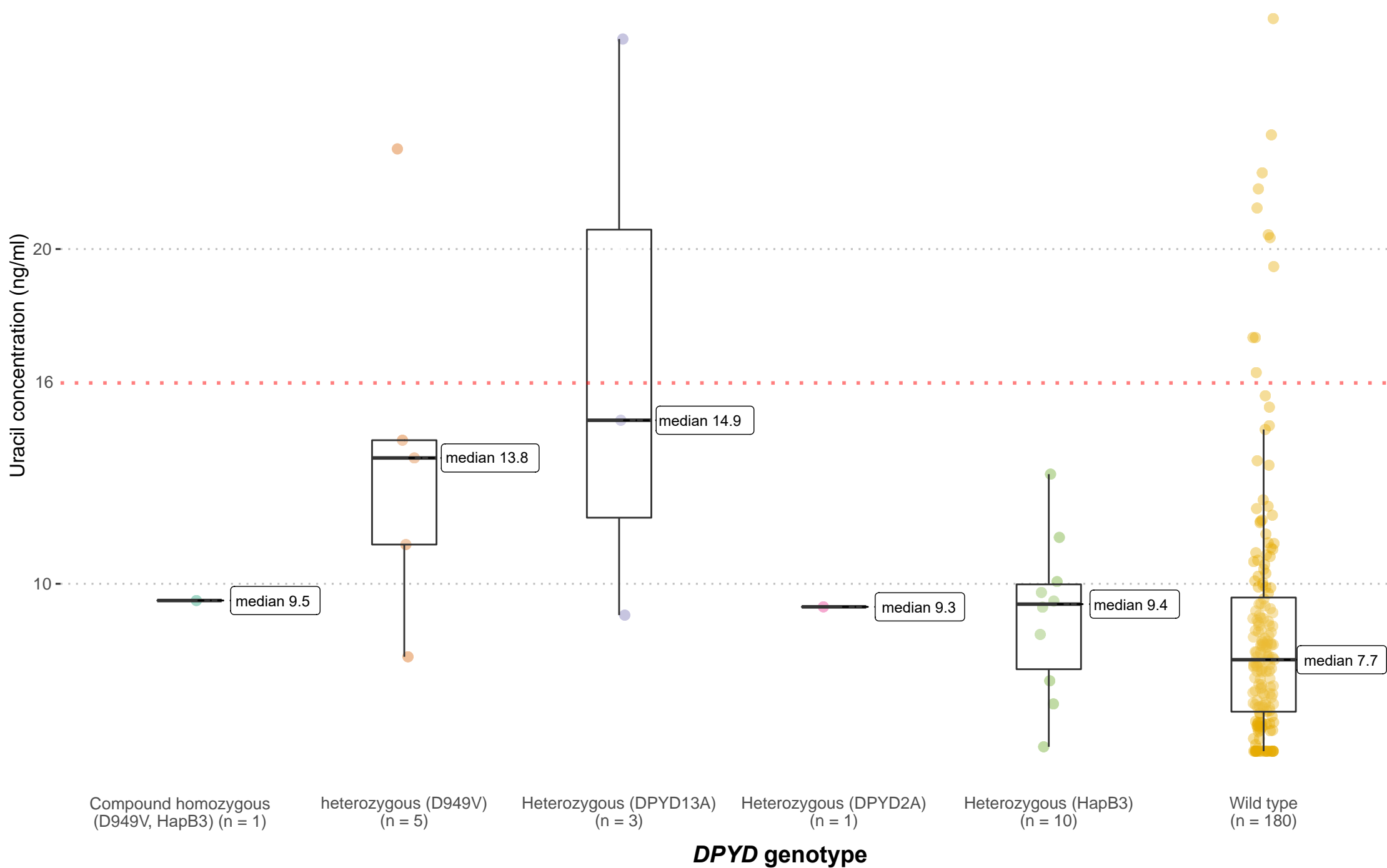

Supplement: Supplementary Figure S4 [file mmc4.pdf]
