# Supplementary material for: Implementation and clinical benefit of DPYD genotyping in a Danish cancer population
Source: ESMO Open. 2023 Feb 13;8(1):100782. doi: 10.1016/j.esmoop.2023.100782 (PMC10024141; doi:10.1016/j.esmoop.2023.100782)

Figure S3. Uracil concentration of *DPYD* variant carriers with grade ≥ 3 FP-related toxicity compared to *DPYD* variant carriers without toxicity. *DPYD* variant carriers was treated with 50% starting dose.

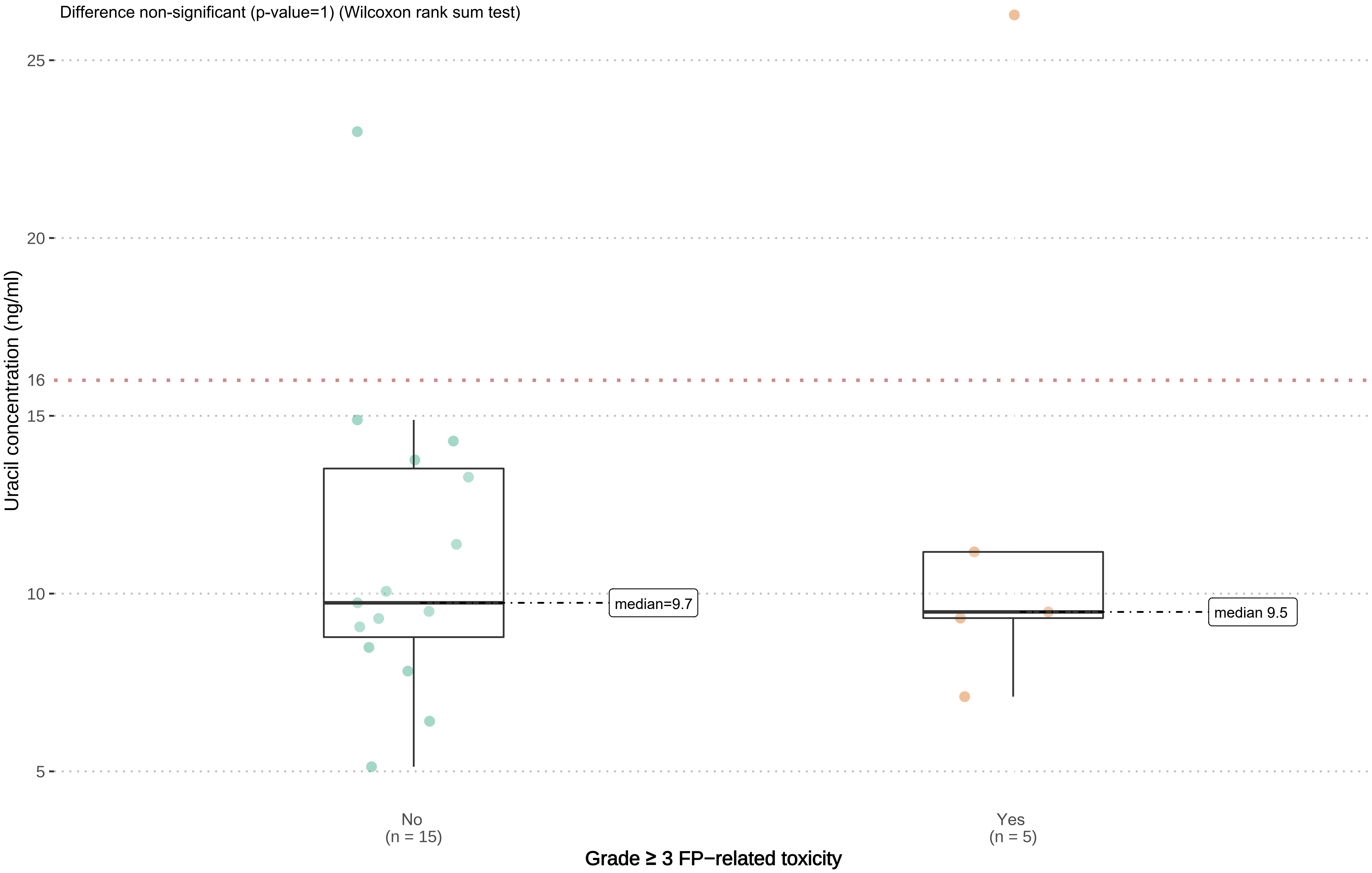

Supplement: Supplementary Figure S3 [file mmc3.pdf]
