# Supplementary material for: Implementation and clinical benefit of DPYD genotyping in a Danish cancer population
Source: ESMO Open. 2023 Feb 13;8(1):100782. doi: 10.1016/j.esmoop.2023.100782 (PMC10024141; doi:10.1016/j.esmoop.2023.100782)
Supplement: Supplementary Table S1, S2, S3 [file mmc5.docx]

**Table S1.**

Frequency of fluoropyrimidine related toxicity in *DPYD*-variant and wild-type patients split into uracil levels.

(heterozygous, compound heterozygous, and homozygous are pooled together)

|  | ***DPYD* variant carriers** | | | **Wild-type patients** | | |
| --- | --- | --- | --- | --- | --- | --- |
|  | Overall,  N = 20 | Uracil <16,  N = 18 | Uracil ≥ 16,  N = 2 | Overall,  N = 180^1^ | Uracil <16,  N = 169 | Uracil ≥ 16,  N = 11 |
| **Relative dose intensity first cycle*** (Minimum-Maximum) | 49%  (38-50)^1^ | 49%  (38-50)^1^ | 50% (50-50) | 96%  (75-100) | 96%  (75-100) | 88%  (75-100) |
| **Overall grade ≥ 3 toxicity** | 5 (25%) | 4 (22%) | 1 (50%) | 53 (29%) | 47 (28%) | 6 (55%) |
| **Stop of FP treatment due to toxicity** | 0 | 0 | 0 | 12 (6.7%) | 10 (5.9%) | 2 (18%) |
| **FP-related hospitalization** | 0 | 0 | 0 | 22 (12%) | 17 (10%) | 5 (45%) |
| **Worst FP grade registered.** |  |  |  |  |  |  |
| **Grade 3 toxicity** | 5 (25%) | 4 (22%) | 1 (50%) | 47 (26%) | 45 (27%) | 2 (18%) |
| **Grade 4 toxicity** | 0 | 0 | 0 | 5 (2.8%) | 1 (0.6%) | 4 (36%) |
| **Grade 5 toxicity (death)** | 0 | 0 | 0 | 1 (0.6%) | 1 (0.6%) | 0 |
| **Grade ≥ 3 gastrointestinal toxicity** | 3 (15%) | 3 (17%) | 0 | 27 (15%) | 23 (14%) | 4 (36%) |
| **Grade ≥ 3 hematological toxicity** | 0 | 0 | 0 | 11 (6.1%) | 7 (4.2%) | 4 (36%) |
| **Grade ≥ 3 cardiac toxicity** | 1 (5.0%) | 1 (5.6%) | 0 | 7 (3.9%) | 7 (4.1%) | 0 |
| **Grade 3 PPE** | 1 (5.0%) | 0 | 1 (50%) | 6 (3.3%) | 6 (3.6%) | 0 |
| **Grade ≥ 3 Other toxicity** | 0 | 0 | 0 | 2 (1.1%) | 0 | 2 (18%) |
| ,*mean in % of normal dose of FP drug relative to regimen. (min-max), All toxicity reported in this table is related to fluoropyrimidines , FP = 5-FU, capecitabine, tegafur(S-1), PPE = Palmar plantar erythrodysesthesia, | | | | | | |

**Table. S2.**

|  | **Intervention group (*DPYD* guided)** | | | | **Control group** | | | |
| --- | --- | --- | --- | --- | --- | --- | --- | --- |
|  | Overall, N = 230^1^ | 5-FU (Fluoruracil), N = 41 | Capecitabine, N = 97 | S-1 (Tegafur), N = 92 | Overall, N = 492^1^ | 5-FU (Fluoruracil), N = 88 | Capecitabine, N = 174 | S-1 (Teysuno), N = 230 |
| **Relative dose intensity first cycle*** | 91%  (38-100) | 94%  (50-100) | 90%  (38-100) | 91%  (50-100) | 98%  (50-100) | 98%  (75-100) | 97%  (50-100) | 98%  (75-100) |
| **Overall grade ≥ 3 toxicity** | 63 (27%) | 9 (22%) | 25 (26%) | 29 (32%) | 112 (23%) | 16 (18%) | 45 (26%) | 51 (22%) |
| **Stop of FP treatment due to toxicity** | 14 (6.1%) | 1 (2.4%) | 5 (5.2%) | 8 (8.7%) | 30 (6.1%) | 5 (5.7%) | 11 (6.4%) | 14 (6.1%) |
| **FP-related hospitalization** | 23 (10%) | 2 (4.9%) | 6 (6.2%) | 15 (16%) | 39 (7.9%) | 6 (6.8%) | 14 (8.0%) | 19 (8.3%) |
| **Worst FP grade registered.** |  |  |  |  |  |  |  |  |
| **Grade 3 toxicity** | 56 (24%) | 9 (22%) | 24 (25%) | 23 (25%) | 94 (19%) | 14 (16%) | 37 (21%) | 43 (19%) |
| **Grade 4 toxicity** | 6 (2.6%) | 0 | 1 (1.0%) | 5 (5.4%) | 12 (2.4%) | 1 (1.1%) | 3 (1.7%) | 8 (3.5%) |
| **Grade 5 toxicity (death)** | 1 (0.4%) | 0 | 0 | 1 (1.1%) | 6 (1.2%) | 1 (1.1%) | 5 (2.9%) | 0 |
| **Grade ≥ 3 gastrointestinal toxicity** | 34 (15%) | 6 (15%) | 8 (8.2%) | 20 (22%) | 60 (12%) | 10 (11%) | 20 (11%) | 30 (13%) |
| **Grade ≥ 3 haematological toxicity** | 11 (4.8%) | 1 (2.4%) | 2 (2.1%) | 8 (8.7%) | 26 (5.3%) | 5 (5.7%) | 5 (2.9%) | 16 (7.0%) |
| **Grade ≥ 3 cardiac toxicity** | 8 (3.5%) | 1 (2.4%) | 5 (5.2%) | 2 (2.2%) | 10 (2.0%) | 1 (1.1%) | 7 (4.0%) | 2 (0.9%) |
| **Grade 3 PPE** | 9 (3.9%) | 0 | 9 (9.3%) | 0 | 14 (2.8%) | 1 (1.1%) | 12 (6.9%) | 1 (0.4%) |
| **Grade ≥ 3 Other toxicity** | 2 (0.9%) | 1 (2.4%) | 0 | 1 (1.1%) | 2 (0.4%) | 0 | 2 (1.1%) | 0 |
| ^1^Mean% (Minimum-Maximum); n (%),*% of normal dose of FP drug relative to regimen. In % (min-max), All toxicity reported in this table is related to fluoropyrimidines, FP = 5-FU, capecitabine, tegafur(S-1), PPE = Palmar plantar erythrodysesthesia, | | | | | | | | |

Frequency of fluoropyrimidine related toxicity in the two groups split into specific drug used.

**Table S3.**

The frequency of fluoropyrimidine-related toxicity in the two groups split into the specific drug used. Only *DPYD*-variant carriers

|  | Only *DPYD*-variant carriers  **Intervention group (*DPYD* guided)** | | | | Only *DPYD*-variant carriers  **Control group** | | | |
| --- | --- | --- | --- | --- | --- | --- | --- | --- |
|  | Overall, N = 22^1^ | 5-FU, N = 4 | Capecitabine, N = 11 | S-1 (Tegafur), N = 7 | Overall, N = 42^1^ | 5-FU , N = 7 | Capecitabine, N = 20 | S-1 (Tegafur), N = 15 |
| **Relative dose intensity first cycle*** | 49%  (38-50) | 50%  (50-50) | 49%  (38-50) | 50%  (50-50) | 97%  (75-100) | 100%  (100-100) | 96%  (75-100) | 99%  (80-100) |
| **Overall grade ≥ 3 toxicity** | 5 (23%) | 1 (25%) | 3 (27%) | 1 (14%) | 12 (29%) | 2 (29%) | 6 (30%) | 4 (27%) |
| **Stop of FP treatment due to toxicity** | 0 | 0 | 0 | 0 | 3 (7.1%) | 1 (14%) | 1 (5.0%) | 1 (6.7%) |
| **FP-related hospitalization** | 0 | 0 | 0 | 0 | 8 (19%) | 1 (14%) | 5 (25%) | 2 (13%) |
| **Worst FP grade registered.** |  |  |  |  |  |  |  |  |
| **Grade 3 toxicity** | 5 (23%) | 1 (25%) | 3 (27%) | 1 (14%) | 7 (17%) | 1 (14%) | 2 (10%) | 4 (27%) |
| **Grade 4 toxicity** | 0 | 0 | 0 | 0 | 3 (7.1%) | 0 | 3 (15%) | 0 |
| **Grade 5 toxicity (death)** | 0 | 0 | 0 | 0 | 2 (4.8%) | 1 (14%) | 1 (5.0%) | 0 |
| **Grade ≥ 3 gastrointestinal toxicity** | 3 (14%) | 1 (25%) | 1 (9.1%) | 1 (14%) | 9 (21%) | 1 (14%) | 4 (20%) | 4 (27%) |
| **Grade ≥ 3 haematological toxicity** | 0 | 0 | 0 | 0 | 4 (9.5%) | 1 (14%) | 3 (15%) | 0 |
| **Grade ≥ 3 cardiac toxicity** | 1 (4.5%) | 0 | 1 (9.1%) | 0 | 0 | 0 | 0 | 0 |
| **Grade 3 PPE** | 1 (4.5%) | 0 | 1 (9.1%) | 0 | 1 (2.4%) | 1 (14%) | 0 | 0 |
| **Grade ≥ 3 Other toxicity** | 0 | 0 | 0 | 0 | 0 | 0 | 0 | 0 |
| ^1^Mean% (Minimum-Maximum); n (%),*% of normal dose of FP drug relative to regimen. In % (min-max), All toxicity reported in this table is related to fluoropyrimidines , FP = 5-FU, capecitabine, tegafur(S-1), PPE = Palmar plantar erythrodysesthesia, | | | | | | | | |
