# Supplementary material for: Implementation and clinical benefit of DPYD genotyping in a Danish cancer population
Source: ESMO Open. 2023 Feb 13;8(1):100782. doi: 10.1016/j.esmoop.2023.100782 (PMC10024141; doi:10.1016/j.esmoop.2023.100782)
Supplement: Appendix [file mmc6.docx]

**Appendix 1**

**Methods**

***DPYD*-genotyping**

Variant alleles of *DPYD*: *2A; rs3918290 (c.1905+1G>A), *13; rs55886062 (c.1679T>G), rs67376798 (c.2846A>T) and HapB3 rs56038477 (c.1236G>A) were genotyped using the LAMP Human DPD deficiency KIT on a LC-GENIE III instrument (LaCAR MDx Technologies, Ougrée, Belgium). Genotyping was performed directly on EDTA-stabilized whole blood. After amplification, melting curve analysis was used to determine the genotypes of each variant using the LC-GENIE III software.

***DPYD-genotyping in the control group***

Genomic DNA was extracted from an aliquot of venous blood with the QIAsymphony DSP DNA Midi kit (Qiagen, Copenhagen, Denmark) using a QIAsymphonyTM SP instrument (Qiagen, Copenhagen, Denmark). Variant alleles of DPYD: *2A; rs3918290 (c.1905+1G>A), *13; rs55886062 (c.1679T>G), rs67376798 (c.2846A>T) and HapB3 rs56038477 (c.1236G>A) were genotyped using predesigned TaqMan SNP genotyping assays: C__30633851_20, C__11985548_10, C__27530948_10 and C__25596099_30 (Applied Biosystems/Thermo Fisher Scientific) in accordance with the manufacturer’s protocol. Real-time PCR was performed using a StepOne Plus real-time instrument (Applied Biosystems, Foster City, California, USA), and genotypes were classified based on TaqMan VIC/FAM intensity values using the auto-call feature of the StepOne Plus Software v2.3.

For comparison of genotyping methods 10 samples were genotyped for *DPYD*-variant alleles (rs3918290, rs55886062, rs67376798, and rs56038477) using both the LC-GENIE III instrument and the StepOne Plus real-time instrument. *DPYD* genotypes determined by both methods for each of the samples were in complete agreement.
***DPD-phenotyping (plasma uracil measurements)***

Briefly, blood was collected before initiating FP treatment or in between treatment cycles and stored in a biobank before analysis. Plasma concentrations were measured by Liquid Chromatography – Tandem Mass Spectrometry (LC-MS/MS). In brief, 300 µl plasma and 20 µl Internal Standard (0.5 µg/ml, 13C14N2-Uracil (Toronto Research Chemicals)) was mixed and 900 µl cold acetonitril was added. After centrifugation, the clear supernatant was collected and evaporated under dry Nitrogen and samples were reconstituated in 100 µl 0.1 % Formic Acid (aq). 25 µl was injected on the chromatographic system (TLX-1 Vanquish (Thermo Scientific)). The analytic column was a Phenomenex Kinetex PS18 2.6µm, 150x3.0 mm. Uracil and Internal Standard was detected using a TSQ Altis (Thermo Scientific) operated in positive electrospray. The analysis was calibrated by in-house prepared calibrators and the relative standard deviation is below 10 %.

**Results**

**Specific FP drugs**

In **supplementary table 1,** the rate of overall grade ≥ 3 FP-TOX is shown for capecitabine, 5-FU, and tegafur (S-1). Overall, no apparent difference in the incidence of overall FP-related toxicity was seen between the three different FP drugs.

As we would expect, the rate of PPE is higher in patients treated with capecitabine than in 5-FU and S-1 (1). No apparent difference in the rate of FP-related cardiac toxicity was observed.

A total of 31 patients were treated with different FP drugs during their first four treatment cycles. Twenty-one of these were changed to another FP drug due to FP-TOX, where the main reasons were: PPE (n=5, capecitabine) and cardiac adverse events (n=4). All four patients in this group experiencing cardiac toxicity were switched to S-1 from 5-FU (n=2) or capecitabine (n=2). Switching to S-1 is known to reduce the risk of recurrence of cardiac toxicity (2).

Only two patients that were switched to another FP drug suffered from ≥ 3-grade toxicity after switching. **Supplementary tabel 2** shows data from only the *DPYD*-variant carriers—no apparent difference in toxicity when comparing 5-FU and capecitabine with S-1, which includes a DPD enzyme inhibitor.

1. Lou Y, Wang Q, Zheng J, Hu H, Liu L, Hong D, et al. Possible Pathways of Capecitabine-Induced Hand–Foot Syndrome. Chemical Research in Toxicology. 2016;29(10):1591–601.

2. Osterlund P, Kinos S, Pfeiffer P, Salminen T, Kwakman JJM, Frödin JE, et al. Continuation of fluoropyrimidine treatment with S-1 after cardiotoxicity on capecitabine- or 5-fluorouracil-based therapy in patients with solid tumours: a multicentre retrospective observational cohort study. ESMO Open. 2022 Jun;7(3):100427.
